# Bi/Mn-Doped BiOCl Nanosheets Self-Assembled Microspheres toward Optimized Photocatalytic Performance

**DOI:** 10.3390/nano13172408

**Published:** 2023-08-25

**Authors:** Shijie Wang, Dongxue Song, Lijun Liao, Bo Wang, Zhenzi Li, Mingxia Li, Wei Zhou

**Affiliations:** 1 Shandong Provincial Key Laboratory of Molecular Engineering, School of Chemistry and Chemical Engineering, Qilu University of Technology (Shandong Academy of Sciences), Jinan 250353, China; wsj0924@qlu.edu.cn (S.W.); lijunliao@qlu.edu.cn (L.L.); zzli@qlu.edu.cn (Z.L.); 2 Key Laboratory of Functional Inorganic Material Chemistry, Ministry of Education of the People’s Republic of China, School of Chemistry and Materials Science, Heilongjiang University, Harbin 150080, China; 13206576070@163.com

**Keywords:** photocatalysis, BiOCl, manganese doping, self-assembly

## Abstract

Doping engineering of metallic elements is of significant importance in photocatalysis, especially in the transition element range where metals possess empty ‘d’ orbitals that readily absorb electrons and increase carrier concentration. The doping of Mn ions produces dipole interactions that change the local structure of BiOCl, thus increasing the specific surface area of BiOCl and the number of mesoporous distributions, and providing a broader platform and richer surface active sites for catalytic reactions. The combination of Mn doping and metal Bi reduces the forbidden bandwidth of BiOCl, thereby increasing the absorption in the light region and strengthening the photocatalytic ability of BiOCl. The degradation of norfloxacin by Bi/Mn-doped BiOCl can reach 86.5% within 10 min. The synergistic effect of Mn doping and Bi metal can change the internal energy level and increase light absorption simultaneously. The photocatalytic system created by such a dual-technology combination has promising applications in environmental remediation.

## 1. Introduction

Semiconductor photocatalysts have drawn considerable attention because of their widespread application in the degradation of organic pollutants in water [1,2,3]. BiOCl, as a classical material with a layered structure, has a promising future in the fields of optics, electricity, and magnetism because of its internal electric field and electron-hole separation resulting from the alternating combination of [Bi_2_O_2_]^2+^ and [Cl^−^] layers [4,5]. However, the performance of BiOCl is limited because it can only be activated by UV light. Therefore, the wider bandgap of BiOCl (about 3.4 eV) necessitates additional methods to improve light utilization. The development of modification techniques for BiOCl groups has been extensively studied, including defect control, construction of heterojunctions, metal deposition, and heteroatom doping. These techniques can also increase the carrier concentration and accelerate the charge transfer rate [6,7,8,9,10].

Among these methods, metal deposition is the compounding of metals on the surface of photocatalysts. It uses the better electrical conductivity of metals to promote electron transfer and effectively prevent electron-hole recombination [11,12]. In addition, due to the different directions of electron transfer, the Schottky barrier or plasma resonance effect will occur, respectively. When the Fermi energy level of metal is lower than that of the semiconductor, electrons tend to transfer from the semiconductor surface to the metal surface. As a consequence, the electron-hole domain is fixed on the metal and semiconductor, respectively, resulting in a Schottky barrier, and thus effectively suppressing the charge recombination [13,14]. When the size of the metal particles is small enough, it is easy to excite hot electrons under visible light irradiation, thus transferring electrons to the semiconductor conduction band position. The collective electron oscillation in the semiconductor conduction band under light irradiation could trigger the surface plasmon resonance effect (SPR) [15,16]. Such a phenomenon can occur not only with precious metals but also with the metal Bi, which has great cost savings and a good overall performance compared to precious metals. Indeed, metal Bi nanoparticle loading has been extensively utilized to improve the photocatalytic performance of semiconductors. For instance, Bi/Bi_2_O_3_ [17], Bi/Bi_2_MoO_6_ [18], Bi/C_3_N_4_ [19], and Bi/CdS [3], etc. photocatalyst systems have been reported. Chang et al. [20] used in situ electron microscopy and theoretical studies to explain in detail the interfacial transport pathways of carriers during the SPR effect induced by metallic bismuth particles in the heterojunction system. Moreover, the metal Bi triggers an increase in the light absorption range after the bandgap change, which is favorable for the photoreactivity of the semiconductor. These studies contribute to a better understanding of the reasons for the increased activity of various bismuth-based photocatalysts.

Heteroatom doping is also a general and effective photocatalyst modification strategy. Because transition metal atoms have unsaturated ‘d’ orbitals, the energy level structure of the semiconductor can be regulated by transition metal atom doping for improved light absorption and photogenerated carrier generation. It can reduce the energy required for electron leap and promote charge separation, thus improving the photocatalytic activity of the photocatalyst [21,22,23,24,25,26]. Currently, numerous researchers have certified that the photocatalytic activity of BiOCl in the degradation of organic pollutants can be improved effectively by metal doping [27,28,29,30]. Manganese (Mn), a common transition metal, is often used for doping into semiconductors because of the diverse valence states of the transition and its low price. Recently, Mn doping has been used to improve the photocatalytic degradation performance of BiOCl. Cen et al. degraded metronidazole using Mn-BiOCl and achieved a removal efficiency of 91.6% after 60 min of treatment under simulated solar light [30]. Pare et al. reported that Mn-BiOCl could remove 98% of malachite green after 120 min under visible light irradiation [31]. In addition, Lin et al. [32] improved the photocatalytic CO_2_ reduction reaction efficiency by doping manganese ions (Mn^2+^) in CsPbBr_3_ halide chalcogenide nanoplates and applying an external magnetic field. Mn doping generates spin-polarized electrons and increases the number of photogenerated carriers using the synergistic effect of Mn doping and the applied magnetic field, which prolongs the carrier lifetime and inhibits charge recombination.

In this work, metal Bi composite and metal Mn doping were used to optimize the bandgap of BiOCl semiconductors. A one-step solvothermal method is used in situ to reduce metal Bi and dope transition metal Mn, both of which modify BiOCl to form Bi/Mn doped BiOCl (MBB) structures. This heterojunction takes advantage of the tight coupling between metallic Bi and BiOCl to shorten the charge transfer distance. Meanwhile, Mn doping changes the internal structure of BiOCl and creates a new bonding energy linkage with it. MBB possesses a higher specific surface area and a more suitable bandgap for light absorption, which enhances the photoresponse and increases the photogenerated carrier concentration and electron-hole separation efficiency. The deposition of metallic Bi and Mn doping modification will further enhance the photocatalytic activity and strengthen the degradation rate of norfloxacin by MBB, thereby constructing a highly active photocatalytic system.

## 2. Experimental Section

### 2.1. Chemicals

Bismuth nitrate pentahydrate (Bi(NO_3_)_3_·5H_2_O) was purchased from Shanghai Dibai Biotechnology Co., Ltd. (Shanghai, China), potassium chloride (KCl) was purchased from Tianjin Guangfu Technology Development Co., Ltd. (Tianjin, China), manganese chloride tetrahydrate (MnCl_2_·4H_2_O) was purchased from Aladdin Reagent Co., Ltd. (Shanghai, China), and *N*,*N*-dimethylformamide (DMF) was purchased from Tianjin Fuyu Fine Chemical Co., Ltd. (Tianjin, China). Norfloxacin (C_16_H_18_FN_3_O_3_) was purchased from Shanghai XianDing Biotechnology Co., Ltd. (Shanghai, China), and sodium sulfate (Na_2_SO_4_) was purchased from Aladdin Reagent Co., Ltd. (Shanghai, China). All the above chemicals are analytical grade and used without further purification.

### 2.2. Synthesis

The Bi/Mn-doped BiOCl microspheres were prepared by a solvothermal method. A total of 6 mmol Bi(NO_3_)_3_·5H_2_O and 6 mmol KCl were added to 32 mL DMF. After stirring for 30 min, a certain amount of MnCl_2_·4H_2_O (molar ratio Mn:Bi = 1:20, 1:30, 1:40) was added and stirred until complete dissolution. The mixture was then transferred to a 50 mL polytetrafluoroethylene (PTFE)-lined stainless steel autoclave for 3 h at 160 °C. The reaction was cooled down and the catalyst was washed with water and ethanol. Finally, the catalyst was dried at 60 °C. The collected sample was denoted as MBB (the molar ratio of Mn:Bi = 1:30 was optimal and this ratio was used for subsequent tests).

Next, 6 mmol Bi(NO_3_)_3_·5H_2_O and 6 mmol KCl were added to 32 mL DMF. After stirring for 30 min, the solution was transferred to a 50 mL PTFE-lined stainless steel autoclave for continuous reaction at 160 °C for 12 h. The collected sample was denoted as Bi/BiOCl.

Then, 6 mmol Bi(NO_3_)_3_·5H_2_O and 6 mmol KCl were added to 32 mL distilled water and the solution was stirred for 30 min. A certain amount of MnCl_2_·4H_2_O (molar ratio Mn:Bi = 1:30) was added, stirred until complete dissolution, and then the mixture was transferred to a 50 mL PTFE-lined stainless steel autoclave for 3 h at 160 °C. The collected sample was denoted as MB.

Finally, 6 mmol Bi(NO_3_)_3_·5H_2_O and 6 mmol KCl were added to 32 mL distilled water, the solution was stirred for 30 min, and the mixed solution was transferred to a 50 mL PTFE-lined stainless steel autoclave for continuous reaction at 160 °C for 12 h. The collected sample was denoted as BiOCl.

## 3. Results and Discussion

### 3.1. Morphology and Microstructure

**Figure 1:**
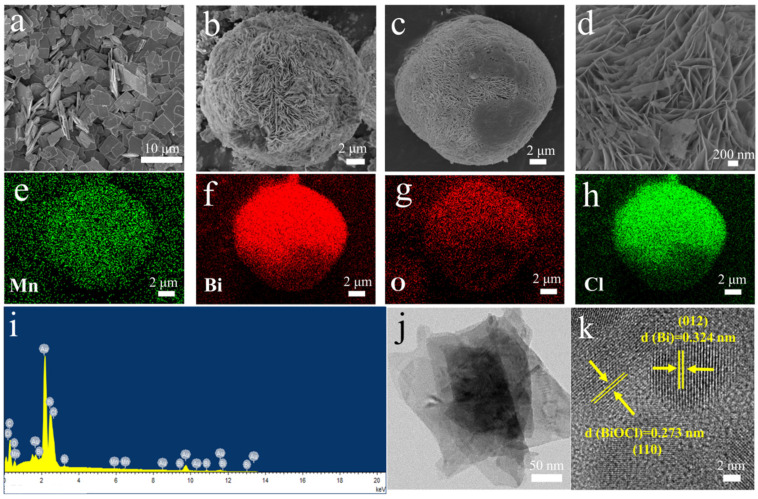
SEM images of BiOCl (**a**), Bi/BiOCl (**b**), MBB (**c**,**d**). Elemental mappings of Mn (**e**), Bi (**f**), O (**g**), and Cl (**h**) in MBB. EDX (**i**), TEM (**j**), and HRTEM (**k**) of MBB.

The scanning electron microscope (SEM) image of Figure 1a demonstrates many distributed BiOCl nanosheets. The nanosheet structure of MB is shown in Figure S1, and the uniform distribution of Mn, Bi, O, and Cl elements in MB nanosheets is shown in Figure S2. The aggregation of nanosheets forms the microsphere structure of Bi/BiOCl as shown in Figure 1b. Figure 1c,d shows the structural features of MBB. It is observed that MBB is formed by the self-assembly of many nanosheets into tightly bound microspheres, and this structure minimizes the agglomeration of nanosheets [22]. The surface free energy of the thinner nanosheets allows them to be uniformly dispersed and combined into a microsphere structure [33], which maximizes the exposure of the specific surface area and increases the active sites for photocatalysis.

Meanwhile, the mapping images (Figure 1e–h) show that each element is uniformly distributed. The presence of Mn elements can be observed in the mapping images [34]. The energy dispersive X-ray (EDX) of Figure 1i demonstrates the distribution of the elements, with the four elements Mn, Bi, O, and Cl co-existing in the MBB structure [35]. The transmission electron microscopy (TEM) of Figure 1j clearly shows the ultrathin nanosheet structure of MBB, and two lattice stripes are observed by further high-resolution TEM (HRTEM) images (Figure 1k). Between these stripes, a lattice spacing of 0.324 nm was obtained by exposing the metallic Bi (012) crystal plane. There is also a lattice spacing of 0.273 nm obtained by exposing the BiOCl (110) crystal plane, which is slightly reduced compared to the original crystal plane spacing, resulting from the effect of lattice distortion caused by Mn doping.

**Figure 2:**
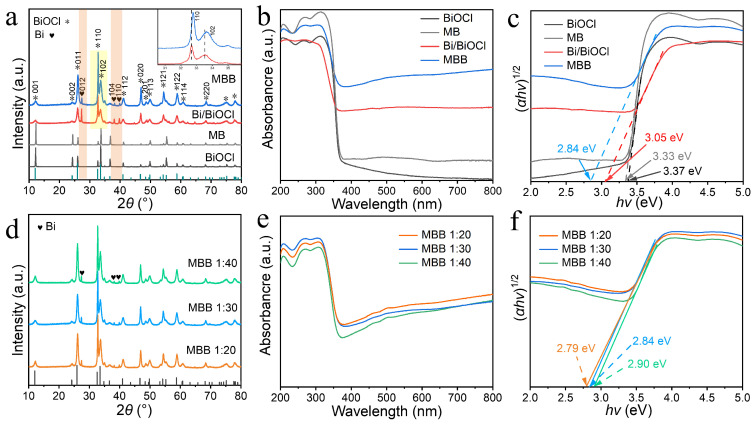
XRD patterns (**a**), UV-vis diffuse reflectance spectra (**b**), and the bandgap (**c**) of BiOCl, MB, Bi/BiOCl, and MBB, respectively. XRD patterns (**d**), UV-vis diffuse reflectance spectra (**e**), and the bandgap (**f**) of MBB (Mn:Bi = 1:20, 1:30, 1:40).

The X-ray diffraction (XRD) patterns of Figure 2a clearly show the diffraction peaks of BiOCl and Bi in MBB. The crystallographic planes corresponding to each diffraction peak are marked in detail in Figure 2a. Among them, BiOCl in MBB is in the tetragonal structured crystalline phase, corresponding to the standard card JCPDS no. 73-2060 [27,28]. The diffraction peaks corresponding to the (110) and (011) crystallographic planes are the most significant, indicating that the crystallographic planes where the microsphere nanosheets are heavily exposed are probably the (110) crystallographic planes. The presence of in situ reduced metal Bi in the system was also demonstrated by XRD. There are obvious diffraction peaks of singlet Bi at 2*θ* = 27.2, 37.9, and 39.6°, corresponding to the crystallographic planes (012), (104), and (110), respectively (JCPDS no. 85-1329) [17]. The diffraction peaks of metallic Bi have been marked by orange shading in Figure 2a. Meanwhile, the magnification of the yellow shaded part shows that the diffraction peak of MBB is shifted to a higher angle than that of Bi/BiOCl. This change is due to the replacement of Bi^3+^ (with a larger ionic radius) by Mn^2+^ (which has a smaller ionic radius), occupying the interstitial sites and causing the lattice to contract. The ultraviolet-visible diffuse reflectance (UV-vis) image in Figure 2b reflects the optical properties of the MBB catalyst. As can be seen from Figure 2b, MBB exhibits higher absorption intensity than Bi/BiOCl, MB, and BiOCl in the 400–800 nm range, indicating that the Mn doping and metal Bi together enhance the sensitivity of BiOCl to light. The bandgap values of 2.84, 3.05, 3.33, and 3.37 eV for MBB, Bi/BiOCl, MB, and BiOCl, respectively [36], are seen in the bandgap diagram of Figure 2c. This indicates that MBB has a forbidden bandwidth more suitable for sunlight absorption for two reasons. Firstly, Mn doping creates an intermediate energy level in the energy band structure of BiOCl. This energy level can be used as a bridge for electron leap to achieve the reduced bandgap effect of BiOCl. Secondly, the metal Bi has a larger absorption rate of light and can enhance the utilization of UV and visible light by BiOCl. The above reasons make MBB absorb a broader range of visible light [37].

Figure 2d–f shows the characterization of the modulated MBB (Mn:Bi = 1:20, 1:30, 1:40) series. It is observed in the XRD plots of Figure 2d that there is almost no change in XRD although the amounts of doped Mn are different. However, the presence of metallic Bi in MBB is confirmed by three strong Bi diffraction peaks. In the UV-vis plot of Figure 2e, the light absorption intensity of the three ratios of MBB does not differ by much, but the bandgap values (Figure 2f) are slightly different. The bandgaps of MBB (1:20, 1:30, 1:40) samples are 2.79, 2.84, and 2.90 eV, respectively [36]. Moreover, the bandgap values become smaller as the amount of Mn doping gradually increases. These results indicate that Mn doping can effectively regulate the light absorption property of BiOCl.

**Figure 3:**
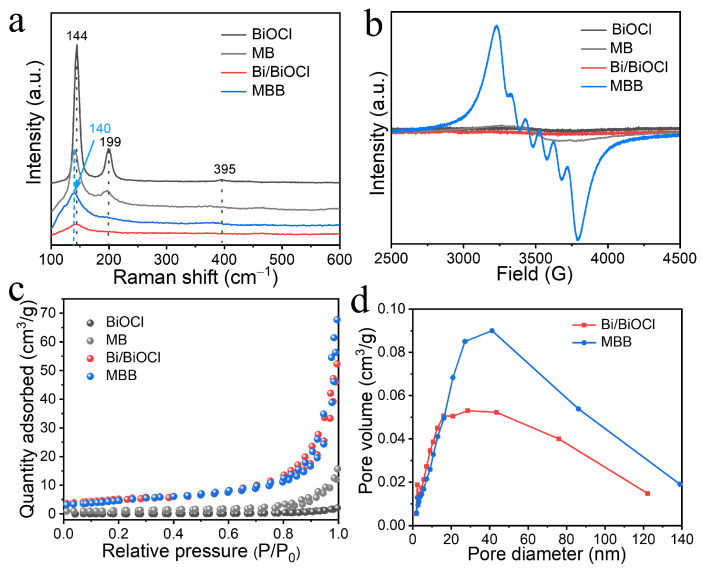
Raman spectra (**a**), EPR (**b**), and N_2_ adsorption–desorption isotherms (**c**) of BiOCl, MB, Bi/BiOCl, and MBB. Pore size distribution curves (**d**) of Bi/BiOCl and MBB.

Figure 3a shows the Raman spectra to determine the vibrational modes of the molecules inside the BiOCl structure. It can be seen from Figure 3a that the characteristic peak of MBB at 144 cm^−1^ caused by the interlayer vibration of *A*_1g_ is significantly weakened and blue-shifted. This difference indicates that the Mn doping in BiOCl may be chemically coordinated with the internal bonds, thus shifting the vibrational peak [38]. The Bi–Cl bond stretching within *E*_g_ occurs at 199 cm^−1^ while the O-atom stretching vibration within *B*_1g_ appears at 395 cm^−1^. Both the above characteristic peaks almost disappear for MBB, indicating that the doping technique and metal composite are effective in modifying the internal structure and surface state of the material [39]. The electron paramagnetic resonance (EPR) pattern in Figure 3b also further demonstrates the doping of Mn elements in BiOCl, showing the stronger signal response of MBB compared to BiOCl, MB, and Bi/BiOCl due to the six EPR vibrational peaks splitting from the interaction between the nuclear spins of Mn^2+^ ions (S = 5/2) and their electron spins. These six splitting peaks indicate that the dipole interaction of Mn^2+^ ions changes the local structure of BiOCl, and indirectly proves the Mn doping in BiOCl [32]. The nitrogen adsorption–desorption isotherm profiles in Figure 3c show typical type IV isotherms for BiOCl, MB, Bi/BiOCl, and MBB, indicating that all three catalysts have a mesoporous structure and MBB exhibits a higher nitrogen adsorption effect. As seen in Table S1, the specific surface area of MBB (21.9 m^2^/g) is not only 1.2 times higher than that of Bi/BiOCl (18.4 m^2^/g), but also 22 times higher than that of BiOCl (1.0 m^2^/g) and 3.4 times higher than that of MB (6.5 m^2^/g). In addition, the pore size pore capacity of MBB is higher than that of Bi/BiOCl, MB, and BiOCl materials. The mesopore distribution curves in Figure 3d also show that MBB has better mesopore distribution peaks, a large number of mesopore structures, and a high specific surface area that can better trap charges, accelerate carrier separation, and provide more active sites for catalytic reactions [40,41].

**Figure 4:**
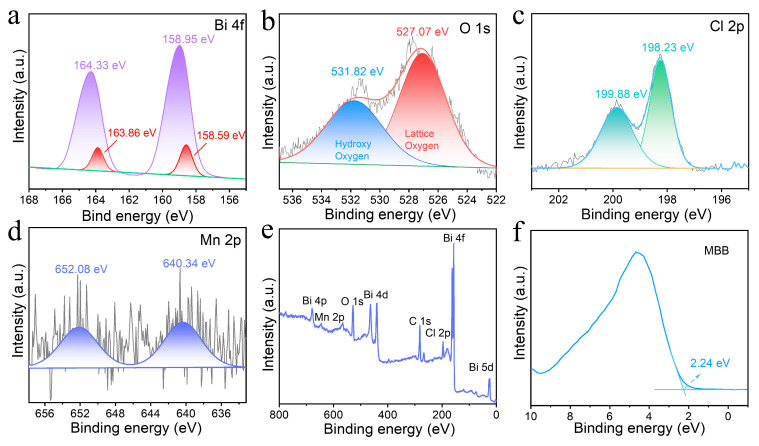
XPS spectra of Bi 4f (**a**), O 1s (**b**), Cl 2p (**c**), and Mn 2p (**d**). Full-scale XPS spectra (**e**) and valence band spectra (**f**) of MBB.

Figure 4a–d shows the X-ray photoelectron spectra (XPS) of the MBB, showing the surface chemical states of the four elements. All elements are calibrated in reference to the binding energy of C 1s (284.8 eV). Figure 4a shows the spectrum of the element Bi in MBB, and the two peaks at 158.95 and 164.33 eV belong to the characteristic peaks of Bi^3+^, corresponding to the Bi 4f_7/2_ and Bi 4f_5/2_ tracks, respectively [42]. In addition, two small peaks at 158.59 and 163.86 eV were separated within the characteristic peak of Bi^3+^, and this peak belongs to the Bi^0^ characteristic peak, which proves the presence of metallic Bi on the surface of the MBB structure. Figure 4b shows the O elemental spectrum in MBB with distinct peaks at 527.07 and 531.82 eV due to metal-O bonding inside the structure and hydroxyl groups on the sample surface, respectively [43]. However, unlike the previous Bi-O characteristic peaks around 529 eV, the MBB binding energy is blue-shifted, probably due to the substitution of the original lattice oxygen in Mn-doped BiOCl to form Mn-O bonds. Figure 4c shows the elemental spectrum of Cl in MBB, and the characteristic peaks at 198.23 and 199.88 eV belong to the Cl 2p_3/2_ and Cl 2p_1/2_ orbitals, respectively. Figure 4d shows the spectrum of the Mn element in MBB. The 640.34 and 652.08 eV split peaks belong to the Mn 2p_3/2_ and Mn 2p_1/2_ orbitals. The combined analysis of XRD, EPR, and XPS speculates that the Mn doping in MBB possesses two forms: one is the replacement of some high-valent Bi^3+^ ions by low-valent Mn^2+^ ions to form Mn-O bonds, which easily induces the vibration of O atoms in MBB, and another is the grafting of Mn^2+^ on the surface of BiOCl to form manganese oxygen clusters, which excites the motion of photogenerated carriers [44,45]. Finally, in the full XPS spectrum of Figure 4e, it can be seen more clearly that the MBB sample contains four elements, Bi, O, Cl, and Mn, thus confirming the doping of Mn. The XPS valence band spectrum energy of MBB is known to be 2.24 eV in Figure 4f. The energy level structure produced by Mn doping can both promote electron leap and block the recombination of photogenerated electrons and holes, thus inhibiting charge recombination.

**Figure 5:**
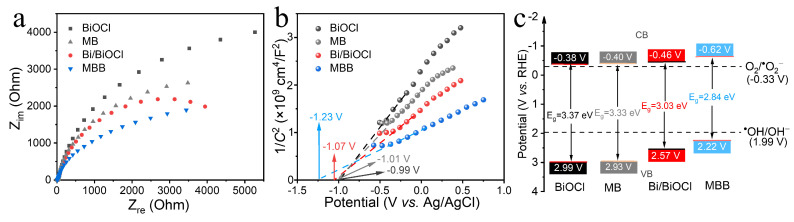
EIS (**a**), Mott–Schottky plots (**b**), and band energy diagram vs. reversible hydrogen electrode (**c**) of BiOCl, MB, Bi/BiOCl, and MBB, respectively.

Figure 5a shows the electrochemical impedance spectrum (EIS) to analyze the resistance ability of the material to the AC current. It can be seen that MBB has a smaller radius of curvature and weaker resistance to current than Bi/BiOCl, MB, and BiOCl, indicating that MBB heterojunction possesses stronger charge transfer ability and diffusion ability [41,46]. The energy level change of MBB can be inferred from the Mott–Schottky curve in Figure 5b, in which the slope of the curves for MBB, Bi/BiOCl, MB, and BiOCl can visually be seen to be positive, reflecting that the materials are all n-type semiconductors. In addition, the extension of the Schottky curve and the intersection of the X-axis determine that the flat-band potentials of MBB, Bi/BiOCl, MB, and BiOCl are −1.23, −1.07, −1.01, and −0.99 V, respectively. The carrier concentration (*N*_d_) in the material can also be derived from Equation (1):

(1) $$N_{d}=\left(\frac{2}{e_{0}\varepsilon \varepsilon_{0}}\right){\left(\frac{d\frac{1}{C^{2}}}{dV}\right)}^{-1}$$

The vacuum dielectric constant (*ε*_0_) of BiOCl is taken as 55 [47]. The carrier concentrations of MBB, Bi/BiOCl, MB, and BiOCl were calculated to be 3.41 × 10^19^, 2.66 × 10^19^, 1.53 × 10^19^, and 1.22 × 10^19^, respectively. MBB exhibits the highest carrier concentrations, showing that it has better conductivity to accelerate the charge flow. Finally, the conduction band positions of the four materials are derived from the flat-band potential, as calculated in Equation (2):

*E* (RHE) = *E* (Ag/AgCl) + 0.0591pH + 0.197 (2)

The calculated CB values for MBB, Bi/BiOCl, MB, and BiOCl are estimated to be −0.62, −0.46, −0.40, and −0.38 V, respectively. Thus, the calculated VB values are estimated to be 2.22, 2.57, 2.93, and 2.99 V based on the equation E_CB_ = E_VB_ − E_g_ [48,49]. Consequently, the involved samples’ energy band structures are determined and shown in Figure 5c.

### 3.2. Photocatalytic Activity and Mechanism

**Figure 6:**
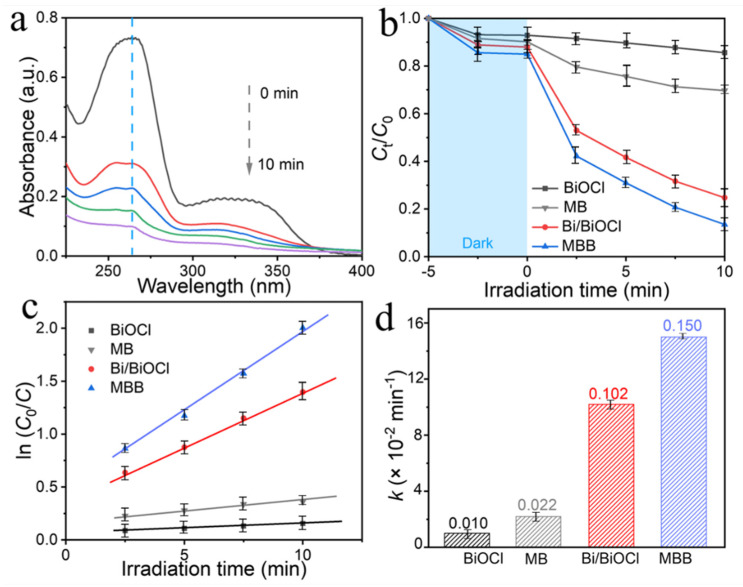
Time-varying absorption spectra of MBB for degradation norfloxacin. The dotted arrow have specified the meaning of the lines from top to bottom. They are in order as follows: 0 min, 2.5 min, 5 min, 7.5 min and 10 min. (**a**). Photocatalytic degradation curves of norfloxacin (**b**), rate curves (**c**), and rate constants (**d**) of BiOCl, MB, Bi/BiOCl, and MBB, respectively.

To further investigate the photocatalytic activity of MBB, photocatalytic degradation of norfloxacin antibiotic was performed to explore its photocatalytic ability. Figure 6a shows the transient time-varying spectra of MBB degradation of norfloxacin. It can be seen that the intensity of the absorbance curve gradually decreases with the increase of illumination time, which indicates that MBB has a significant degradation ability of norfloxacin. The maximum absorbance wavelength of norfloxacin is taken at 264 nm [50]. To investigate the difference in the degradation ability of MBB, Bi/BiOCl, MB, and BiOCl, we clearly show the comparison of the degradation performance of the four materials in Figure 6b. The degradation did not change significantly in the dark treatment. Still, in only 10 min under light, MBB degraded norfloxacin by 86.5%, which was 11.3% (1.15 times) higher than Bi/BiOCl, 56.2% (2.85 times) higher than MB, and 72.1% (6.01 times) higher than BiOCl, respectively. The reason for such a significant photocatalytic performance possessed by MBB is attributed to the interfacial engineering of metal Bi with BiOCl and the Mn-doped structure defect engineering.

Meanwhile, the rate curves of MBB, Bi/BiOCl, MB, and BiOCl conformed to the first-order kinetic equation (Figure 6c), and the rate constants of MBB, Bi/BiOCl, MB, and BiOCl (Figure 6d) were estimated to be 0.150, 0.102, 0.022, and 0.010 min^−1^, respectively. The rate constant of MBB was calculated to be 1.47 times, 6.82 times, and 15 times higher than those of Bi/BiOCl, MB, and BiOCl, respectively. This result more obviously shows that the degradation activity of MBB is higher, which once again proves that Mn doping and metal composite increase the utilization of light, accelerate charge separation and transfer, and improve the degradation rate. The unsaturated ‘d’ orbitals of Mn absorb a large number of electrons, thereby increasing the carrier concentration on the semiconductor surface to further oxidize the contaminant [37,51]. The in situ deposition of metallic Bi and the tightly coupled interface accelerates charge separation. Meanwhile, it further proves that MBB has great potential in environmental remediation, and such a dual technology combination of surface modification and internal structure doping also provides good ideas for the future development of photocatalysts.

In order to study the stability of the MBB photocatalyst, a long-performance test was executed on photocatalytic norfloxacin degradation. As can be seen in Figure S3a, no obvious decrease in degradation rate is observed after four cycles. Furthermore, XRD patterns of MBB hybrid before and after four cycles of photocatalytic degradation reaction were recorded and shown in Figure S3b. It can be clearly seen that the crystal structure of the MBB hybrid does not display a significant change after the long-performance test. These results demonstrate the good stability of the MBB photocatalysts.

**Figure 7:**
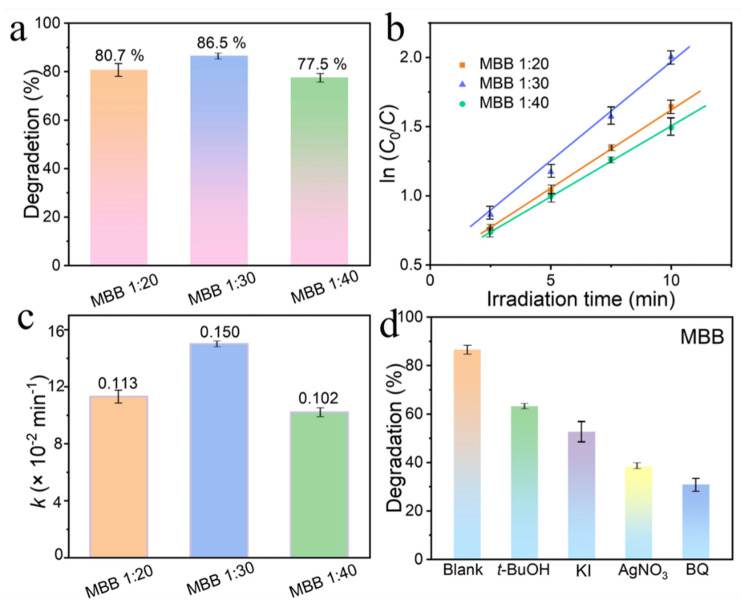
Histograms of photocatalytic degradation rates (**a**), rate curves (**b**), and rate constants (**c**) of MBB (Mn:Bi = 1:20, 1:30, 1:40). The inhibition of norfloxacin degradation by free radical scavengers in the MBB system (**d**).

To understand the effect of Mn doping concentration on the photocatalytic degradation rate of MBB, we investigated different doping concentrations of MBB (Mn:Bi = 1:20, 1:30, 1:40) for the photodegradation of norfloxacin antibiotics. As shown in Figure 7a, the degradation rates of the three samples were 80.7%, 86.5%, and 77.5%, respectively. It can be seen that the best photocatalytic degradation performance was obtained for MBB (1: 30) with different ratios of modulated Mn doping. Such peak changes were also reflected in the rate curves and rate constants. The rate curves in Figure 7b demonstrate that the compound first-order kinetic curve changes for all three samples. It can be estimated that the kinetic constants for MBB (Mn:Bi = 1:20, 1:30, 1:40) were 0.113, 0.150, and 0.102 min^−1^ (Figure 7c). In summary, MBB (1: 30) has the most suitable Mn doping concentration and metal Bi complex, which allows MBB to degrade norfloxacin at optimal performance. Usually, the photocatalytic degradation process occurs with three reactive species ^•^OH, ^•^O_2_^−^, and h^+^ in the degradation system, as shown in Figure 7d. Each of the three substances is added to play an inhibitory role in the degradation process to identify the radicals that play a major and minor degradation role in the reaction system, with the capture of hydroxyl groups, hole, electrons, and superoxide by tert-butanol (*t*-BuOH), potassium iodide (KI), silver nitrate (AgNO_3_), and benzoquinone (BQ), respectively [52]. Figure 7d shows that adding AgNO_3_ and BQ has a stronger inhibitory effect on the reaction system, indicating that the magnitude of radical activity in the MBB degradation system is ^•^O_2_^−^ > h^+^ > ^•^OH. The reaction system is characterized by ^•^O_2_^−^ is the main active species; h^+^ and ^•^OH are the secondary active species.

**Figure 8:**
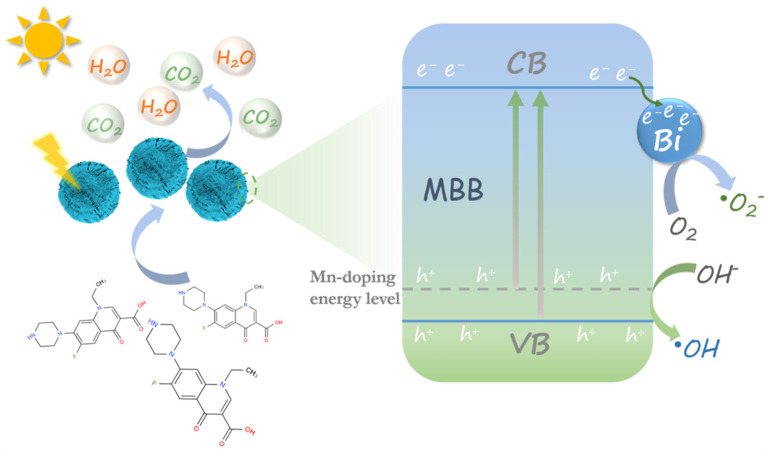
The proposed photocatalytic mechanism of MBB heterojunction assemblies.

As the mechanism diagram presented in Figure 8, MBB is a microsphere formed by a large number of nanosheets because of the surface free energy assembly. A Mn 3d-O 2p intermediate energy level formed near the top of the valence band of BiOCl in its energy level via Mn doping. The introduced Mn 3d-O 2p intermediate energy level narrowed the bandgap of BiOCl, optimizing light absorption and modulating the photoelectric properties of BiOCl. Moreover, abundant OVs caused by Mn-doping would serve as electron traps to promote the separation of photogenerated carriers [53,54]. The tightly coupled interface of metal Bi and the Mn doping within the structure together broadens the light absorption range, enhances the carrier lifetime, and accelerates the carrier transfer, which shows the degradation of highly toxic norfloxacin; sunlight irradiation can convert the antibiotics into CO_2_, H_2_O, and other non-toxic and harmless small molecules. Meanwhile, the energy level structure of MBB excites charge transfer from VB to CB under illumination, leaving holes at the VB position. The surface engineering of MBB is demonstrated by the interfacial coupling of metal Bi, which allows electrons from the CB of BiOCl to leap to the Bi surface to increase the electron concentration. Electrons concentrated on the surface of metal Bi can convert the O_2_ adsorbed on the catalyst surface into •O_2_^−^ [37]. Furthermore, the internal engineering of MBB is achieved by Mn doping to change the energy level position. The holes in the VB position can convert OH^−^ in water to •OH. In summary, both tight interfacial coupling techniques and energy-level structure engineering are important in creating high-performance catalysts. Both in situ reduced metal Bi and internally doped Mn contribute to the high catalytic activity of MBB and build up the complete photocatalyst system.

## 4. Conclusions

In conclusion, the MBB microsphere structure was constructed by a simple solvothermal method, using in situ reduced metallic Bi to form a tight heterogeneous interface and reduce the charge separation resistance. Replacing high-valent Bi atoms with low-valent Mn to create Mn doping can build an intermediate energy level and accelerate electron transfer. The metal Bi and the charged defect generated by Mn doping together promote the utilization of the light by BiOCl. The specific surface area of MBB and active sites were also increased to promote the photocatalytic degradation of norfloxacin. The degradation rate of MBB was 1.15 times that of Bi/BiOCl, 2.85 times that of MB, and 6 times that of BiOCl. Furthermore, the rate constant of MBB was 1.47 times, 6.82 times, and 15 times higher than those of Bi/BiOCl, MB, and BiOCl, respectively. Such efficient photocatalytic activity can be ascribed to in situ anchored metal Bi and Mn doping with unsaturated ‘d’ orbitals. These modifications greatly promote the charge transfer within the MBB structure and prolong the carrier lifetime. Superoxide anions are the main active substances confirmed by the capture experiments. The combination of two structural engineering techniques can be used to create efficient photocatalysts to provide a broader range of ideas for future catalyst modification.

## Data Availability

No additional data are available.

## Supplementary Materials

The following supporting information can be downloaded at: https://www.mdpi.com/article/10.3390/nano13172408/s1, Figure S1: SEM image of MB; Figure S2: Elemental mappings of Mn (a), Bi (b), O (c), and Cl (d) in MB; Figure S3: Cycling runs for the photocatalytic degradation of norfloxacin in MBB nanocomposite suspensions. (a); (b) XRD patterns of MBB before and after photocatalytic degradation reaction for four cycles (b); Table S1: The specific surface areas, pore diameters, and pore volumes for BiOCl, MB, Bi/BiOCl and MBB, respectively.
