# Supplementary material for: Bi/Mn-Doped BiOCl Nanosheets Self-Assembled Microspheres toward Optimized Photocatalytic Performance
Source: Nanomaterials (Basel). 2023 Aug 25;13(17):2408. doi: 10.3390/nano13172408 (PMC10490148; doi:10.3390/nano13172408)
Supplement: Supplementary file 1 [file nanomaterials-13-02408-s001.zip › nanomaterials-2524116-supplementary.pdf]

Supporting Information

Bi/Mn-Doped BiOCl Nanosheets Self-Assembled Microspheres toward Optimized Photocatalytic Performance

S1. Experimental section

S1.1. Characterizations

Powder X-ray diffraction (XRD) patterns were analyzed by Bruker D8 Advance diffractometer with monochromatic Cu Kα Radiation (λ = 1.5406, acceleration voltage 40 kV, applied current 20 mA). Raman spectra were presented by Jobin Yvon HR 800 micro Raman spectrometer at 457.9 nm. The refined structure and morphology of the samples were recorded on scanning electron microscopy (SEM, FEI Sirion 200 instrument operated at 15 kV) and transmission electron microscopy (TEM, Tecnai G2 F20, acceleration voltage 200 kV). By X-ray electron spectroscopy (XPS), Al Kα (1253.6 eV) was studied Super axial DLD surface state of the achromatic X-ray source. An ultraviolet-visible spectrophotometer (λ950 (Perkin Elmer, USA)), diffuse reflectance spectroscopy (DRS) was performed in the range of 200-800 nm, with barium sulfate fine powder as the reference. The nitrogen adsorption-desorption isotherms at 77 K were collected on the AUTOSORB-1 (Quantachrome instrument) nitrogen adsorption instrument. The Bruner Emmett Taylor equation is used to estimate the specific surface area. The pore size distribution was measured using Barrett–Joyner–Halenda (BJH) measurements from the isotherm adsorption branch. X-band electron paramagnetic resonance (EPR) was recorded using BRUKER N500 electron paramagnetic resonance spectrometer.

S1.2. Photocatalytic activity measurements

The photocatalytic activity of norfloxacin ($C_{16}H_{18}FN_3O_3$) was tested by its photocatalytic degradation reaction. An AM 1.5 solar power system with a 300 W cut-off filter Xe lamp with an AM 1.5 filter was used as the light source. The calibrated light intensity was 100 MW cm^{-2} before the performance

measurements. Typically, the prepared photocatalyst (50 mg) was added to 50 mL of norfloxacin solution (10 mg/L). To achieve adsorption-desorption equilibrium, the suspension was left to stand in the dark at room temperature for 5 min. Then, the suspension was irradiated with high-speed stirring and 4 mL of the solution was taken every 5 min and filtered through a 0.22 μm microporous filter to filter the photocatalyst from the solution. The UV-Vis absorption spectra of the residual norfloxacin were recorded with a UV-Vis spectrophotometer (Shanghai Mepro Delta UV-1800 BPC).

S1.3. Photoelectrochemical measurements

The photoelectrochemical performance test was performed with Ag/AgCl as reference electrode, platinum sheet as a counter electrode, FTO glass that has been sprayed with MBB as photoanode, 0.2 M Na_2SO_4 solution as electrolyte (the solution needs to be used after passing nitrogen gas), and finally a three-electrode system with an electrochemical workstation (Princeton Versa STAT 3) for electrochemical performance analysis. The electrochemical test conditions were as follows: alternating current (AC) was set at a starting frequency of 10,000 Hz, an ending frequency of 0.1 Hz, and an overvoltage of 600 mV. The photoanode was prepared using the conventional spray method: 30 mg of sample was added to 8 mL of ethanol and the suspension was dispersed homogeneously by ultrasonication. The uniformly dispersed MBB solution was then sprayed onto the transparent FTO glass surface (TCO, fluorine doped tin oxide layer, Japanese flat glass) with a spray gun. The FTO glass coated with MBB was calcined at 350 °C in N_2 at a heating rate of 5 °C/min for 2 h. In the photoelectrochemical test, the coating area and illumination area were 1.5 cm^2 and AM 1.5 power system was used as the light source.

S2. Supporting Figures and Table

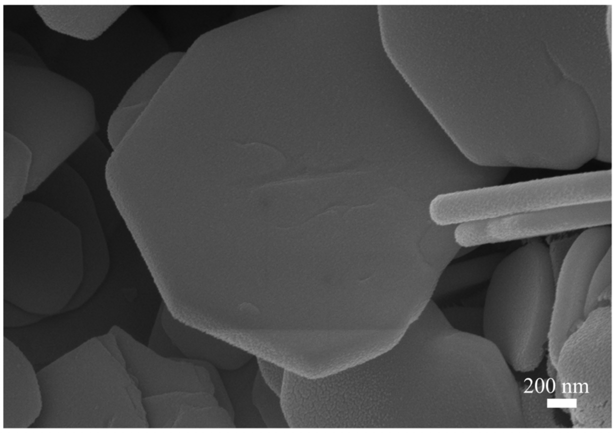

Figure S1. SEM image of MB.

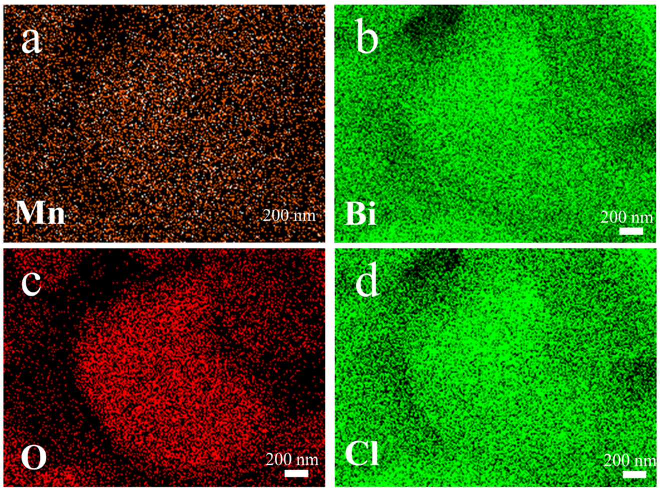

Figure S2. Elemental mappings of Mn (a), Bi (b), O (c), and Cl (d) in MB.

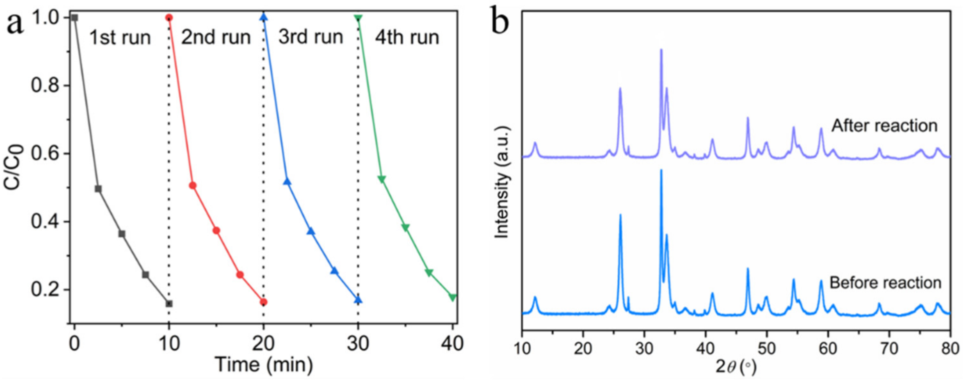

Figure S3. Cycling runs for the photocatalytic degradation of norfloxacin in MBB nanocomposite suspensions. (a); (b) XRD patterns of MBB before and after photocatalytic degradation reaction for four cycles (b).

Table S1. The specific surface areas, pore diameters, and pore volumes for BiOCl, MB, Bi/BiOCl and MBB, respectively.

Sample	Surface Area (m^2g^{-1})	Pore Size (nm)	Pore Volume (cm^3g^{-1})
BiOCl	1.0	16.6	0.002
MB	6.5	16.8	0.009
Bi/BiOCl	18.4	18.9	0.070
MBB	21.9	24.0	0.094
